# Viral and Non-Viral Systems to Deliver Gene Therapeutics to Clinical Targets

**DOI:** 10.3390/ijms25137333

**Published:** 2024-07-04

**Authors:** Maryam Taghdiri, Claudio Mussolino

**Affiliations:** 1 Institute for Transfusion Medicine and Gene Therapy, Medical Center—University of Freiburg, 79106 Freiburg, Germany; 2 Center for Chronic Immunodeficiency (CCI), Medical Center—University of Freiburg, 79106 Freiburg, Germany; 3 Ph.D. Program, Faculty of Biology, University of Freiburg, 79106 Freiburg, Germany; 4 Faculty of Medicine, University of Freiburg, 79106 Freiburg, Germany

**Keywords:** delivery, gene therapy, lipid nanoparticles, non-viral delivery, lentiviral vectors

## Abstract

Clustered regularly interspersed short palindromic repeats (CRISPR)/CRISPR-associated protein 9 (Cas9) technology has revolutionized the field of gene therapy as it has enabled precise genome editing with unprecedented accuracy and efficiency, paving the way for clinical applications to treat otherwise incurable genetic disorders. Typically, precise genome editing requires the delivery of multiple components to the target cells that, depending on the editing platform used, may include messenger RNA (mRNA), protein complexes, and DNA fragments. For clinical purposes, these have to be efficiently delivered into transplantable cells, such as primary T lymphocytes or hematopoietic stem and progenitor cells that are typically sensitive to exogenous substances. This challenge has limited the broad applicability of precise gene therapy applications to those strategies for which efficient delivery methods are available. Electroporation-based methodologies have been generally applied for gene editing applications, but procedure-associated toxicity has represented a major burden. With the advent of novel and less disruptive methodologies to deliver genetic cargo to transplantable cells, it is now possible to safely and efficiently deliver multiple components for precise genome editing, thus expanding the applicability of these strategies. In this review, we describe the different delivery systems available for genome editing components, including viral and non-viral systems, highlighting their advantages, limitations, and recent clinical applications. Recent improvements to these delivery methods to achieve cell specificity represent a critical development that may enable in vivo targeting in the future and will certainly play a pivotal role in the gene therapy field.

## 1. Introduction

Genetic diseases are due to mutations that impair gene functions, ultimately leading to dysfunctional cells and tissues. Gene therapy aims at using the transfer of genetic material into the diseased cells in order to heal them [1]. In the area of blood disorders, the ability to modify patient’s own hematopoietic stem cells ex vivo prior to retransplantation has paved the way for the clinical use of gene therapeutics for a variety of human conditions, including primary immunodeficiencies, hemoglobinopathies, and metabolic defects [2,3]. Initial strategies typically employed recombinant viral vectors to deliver a copy of the defected gene to the target cells and reconstitute the missing gene function. While this is a viable strategy in the case of recessive genetic disorders, in the context of dominant conditions, the simple overexpression of the healthy gene might not be sufficient. Precise genome editing offers an alternative as it allows the correction of the underlying mutation using a variety of approaches that exploit cellular DNA repair mechanisms to install specific genetic alterations [4]. In the last two decades, multiple technologies have been developed and refined for this purpose. With the recent addition of CRISPR-Cas9 to the toolbox of gene editors, the field of genetic engineering has been revolutionized by rendering genome engineering approaches accessible to more and more laboratories worldwide [5]. As a consequence, continuous refinement and enhancement of these platforms have profoundly impacted the field of gene therapy, shaped by the promise of providing therapeutics for virtually any genetic disorder. It has soon become evident that a critical aspect of success is the ability to deliver all the components necessary to modify the target cells without compromising cell viability. These include the delivery of DNA, RNA, or proteins that have to overcome multiple biological barriers in order to be available within the cells for the final editing process. Different strategies have been developed to deliver gene therapies, either gene therapeutics or precise genome editing components, into transplantable cells. The use of viral vectors has pioneered the field, enabling the manufacturing of gene therapeutics following gene replacement strategies for clinical purposes. Several primary immunodeficient patients have been successfully cured by receiving a transplant of their own hematopoietic stem cells after transduction ex vivo with integrating viral vectors harboring a correct copy of the mutated disease-causing gene, eventually complementing the missing function [6]. Despite the success, critical challenges have been identified, such as the potential risks associated with the vector integration, the manufacturing costs of the vector itself and ultimately of the therapeutic product, as well as the difficulties of adapting such an approach for direct in vivo editing [7]. These have prompted the establishment of novel and more precise strategies to attempt to tackle these challenges by achieving targeted gene modifications.

More recently, non-viral systems have been used to genetically engineer target cells ex vivo. Clinical exploitation of these strategies has highlighted the potential of gene therapy applications but also the challenge of improving delivery methods in order to facilitate manufacturing and possibly reduce the costs of this type of therapeutic. The implementation of technologies that enable cell-specific delivery is paving the way for direct in vivo delivery, which has shown remarkable potential thus far and will certainly further simplify the gene therapy procedure. Here, we describe the major delivery methods available and highlight their clinical implementation, with a focus on those that enable in vivo delivery.

## 2. Delivery Carriers for Gene Therapy

### 2.1. Viral Vectors

**Figure 1:**
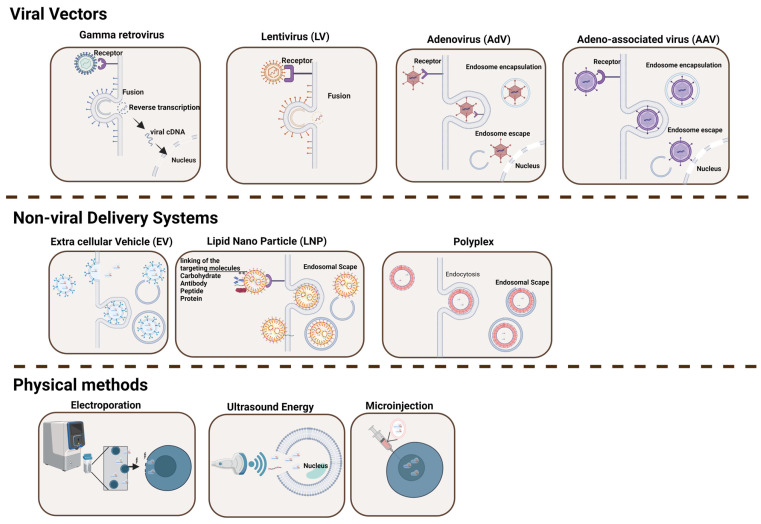
Delivery Systems for Gene Editing Components. This figure illustrates the diversity of delivery tools and methodologies utilized in gene therapy to shuttle a defined cargo into target cells. The various types of delivery methods described in the text are indicated, including viral vectors (gamma retrovirus, lentivirus, adenovirus, and adeno-associated virus), physical methods (ultrasound energy, microinjection, and electroporation), and non-viral delivery systems (extracellular vesicles, lipid nanoparticles, and polyplex) [10,11,12,13,14]. While viral vectors typically offer high transduction efficiency, they often provoke immune responses and have cargo limitations. In contrast, non-viral approaches are generally safer and more versatile but may exhibit lower efficiency. Figure created with BioRender.com.

Viral vectors have naturally evolved to release their genome into target cells and therefore have been engineered for a variety of purposes, including gene delivery and vaccines (Figure 1 and Table 1) [8]. The proteins that constitute the surface of the virus are typically capable of overcoming cellular physical barriers, thus allowing the deposition of their cargo (i.e., DNA, mRNA, and other materials) effectively inside the target cell [9]. Four major types of recombinant viral vectors have been used in the gene therapy field in the last few decades. These include gamma retrovirus (γ-RV), lentivirus (LV), adenovirus (AdV), and adeno-associated virus (AAV).

Gamma retroviruses are enveloped RNA viruses that, once in contact with the target cells, are capable of introducing their genome into the cytoplasm, where it is retro-transcribed to double-stranded DNA prior to integration into the host genome [32]. For this reason, these viruses guarantee sustained expression of the genetic information they harbor, and they have been largely used both as molecular biology tools and in clinical applications. Their large genome size allows the efficient transfer of up to 10 kb of genetic material to the host cell, and this has been exploited to deliver a variety of therapeutic genes to transplantable cells to develop innovative gene therapies. Initially, recombinant retroviral vectors used either natural or synthetic viral elements to drive high transgene expression [33]. The first clinical applications used these vectors to restore the missing gene function in hematopoietic cells from patients affected by severe combined immunodeficiency-X1 (SCID-X1) [34]. However, soon after retransplantation of the modified cells, it appeared evident that the integration capacity of retroviruses has a critical safety concern as it might result in insertional mutagenesis and uncontrolled proliferation, as occurred in a consistent number of patients from this trial (Table 2) [35]. Interestingly, the same vector used in a different condition, namely the adenosine deaminase deficient severe combined immunodeficiency (ADA-SCID), was very successful in providing benefit to patients. The treatment has received market authorization under the name of Strimvelis (Table 3), and only a single adverse case has been reported after over 10 years of follow-up, despite a similar integration profile as seen in the SCID-X1 trial [36]. The lesson learned in this case led to modifications of the viral genome to mitigate this problem by inactivating those elements that could drive unregulated expression of the genes in close proximity to the virus integration site [37]. The so-called self-inactivating (SIN) retroviral vectors have since been used, but mostly in non-clinical applications, as they have been largely replaced in clinics by the more effective lentiviral vectors with the same SIN configuration.

Lentiviruses also belong to the genus of retroviruses and possess two copies of a single-stranded RNA genome. The basic structure of LVs, which has been largely applied in basic and clinical applications, is based on the human immunodeficiency virus type 1 (HIV-1) and, in contrast to gamma retroviruses, it can infect both dividing and non-dividing cells as its genome is capable of crossing the nuclear membrane [44]. This feature has paved the way for applications aimed at modifying quiescent cell types, such as neurons, and, together with their limited immunogenicity, has contributed to the widespread use of this system for a variety of pre-clinical and clinical applications. Even though lentiviruses have a slightly reduced preference to integrate in proximity to gene transcription start sites as compared with gamma-retroviruses [45], they share a similar integration mechanism and therefore entail the same concerns of insertional mutagenesis when used as viral vectors, as mentioned above (Table 2). The adoption of the SIN strategy, as seen for retroviral vectors, has significantly improved their safety, with no reported case of cell transformation in an ongoing clinical trial using these vectors as of today [46,47,48,49]. To further improve safety, integrase-defective lentiviral vectors (IDLV) have been developed with the goal of reducing viral genome integration by introducing specific mutations in the viral integrase gene [50]. This approach has enabled the use of LV-mediated gene transfer for transient expression of exogenous transgenes [51,52]. This enhancement has been pivotal in the genome engineering field as it has allowed the efficient delivery of designer nucleases, such as the CRISPR-Cas9 system, in transplantable cells where sustained nuclease expression is generally unwanted due to its potential genotoxicity [53]. Comparably, the IDLV platform has been largely used to deliver repair template sequences for genome editing, further expanding the use case of this platform [54]. These improvements have contributed to making the LV platform the most used system in the gene therapy and gene editing fields and have enabled marketing approval for medicinal products based on transplantable cells modified using these vectors (Table 3) [55,56,57,58,59]. Similarly, the same vector is also effectively used to modify patient-derived T cells to armor them with chimeric antigen receptors to specifically recognize and kill cancer cells [60].

Adenoviruses are non-enveloped and non-integrating viruses that harbor a double-stranded DNA genome of about 36 kb. AdV can infect both dividing and non-dividing cells and, given their large genome size, offer more flexibility in the size of exogenous DNA that can be accommodated for transfer as compared with retroviral and lentiviral vectors [61]. First-generation AdV vectors can deliver approximately 8.5 kb of cargo, as only those viral genes responsible for replication have been deleted [62,63]. However, the third AdV vector generation, also known as helper-dependent or gutless, has most of its genome deleted and retains only the inverted terminal repeats and the packaging signal, which are essential for DNA replication and encapsulation. This leaves large space for transgene delivery [64]. Importantly, this virus shows only a modest level of integration into the host genome, therefore being less susceptible to insertional mutagenesis concerns than the other vectors described above [65]. The large amount of knowledge accumulated in early clinical trials has rendered this type of viral vector one of the best studied, with a tremendous amount of data collected that has contributed to understanding their safety. However, the large number of patients treated with this vector has highlighted a non-trivial immunogenicity due to the vector itself, consequent to the large prevalence of AdV-specific antibodies in the human population (Table 2) [66]. On the one hand, this knowledge has been exploited to generate potent AdV-based cancer therapeutics [67], but on the other hand, it has contributed to a critical step back in the whole gene therapy field when AdV-induced immune response caused the death of a patient enrolled in a trial for the treatment of ornithine transcarbamylase [68]. Numerous approaches have been proposed to reduce the consequences of AdV immunity. These include the use of viruses with low seroprevalence, the use of non-human AdV vectors, copolymer encapsulation in modified AdVs, and manipulation of the vector genome to reduce immunogenicity and diminish undesirable surface interactions [69,70,71,72]. In most cases, these alternative strategies have been shown to be less potent, and further studies are necessary to appreciate their potential. In the genome editing field, however, AdV vectors offer the unique opportunity to deliver large transgenes, such as designer nucleases, in a transient manner, thus enabling precise genome editing approaches directly upon in vivo administration [73]. In particular, in recent years, AdV has been exploited to deliver the CRISPR-Cas9 system with the goal of exploring novel therapeutic strategies. Effective restoration of dystrophin expression has been achieved by direct delivery of an AdV harboring CRISPR-Cas9 to excise the mutant exon 23 in a Duchenne muscular dystrophy mouse model [74]. More complex editing relying on homologous directed repair has also been successfully demonstrated by delivering all editing components using AdVs to correct the hemophilia B defect in mice [75]. As these studies also highlighted the activation of adaptive immunity against the virus and Cas9, a proper understanding of the safety concerns is crucial for their successful application.

Adeno-associated virus (AAV) is a small, non-enveloped, single-stranded DNA virus belonging to the *Parvoviridae* family. It is classified as a *dependovirus* as it lacks critical genes necessary for replication, which have to be provided by other viruses such as AdV [76]. The AAV genome is a single-stranded DNA fragment of about 4.7 kb protected by a capsid made up of three virally encoded structural proteins, VP1, VP2, and VP3, assembled in a defined 1:1:10 ratio [77]. This virus is capable of infecting a variety of human cells as a consequence of variable capsid properties that have been studied among hundreds of variants isolated from living organisms. These have been grouped into thirteen classes, each with a specific tropism and depending on defined host cell receptors for entry [78]. Although the high prevalence of serum antibodies against different AAV serotypes indicates that most individuals have been infected at least once in their lives by an AAV and there is evidence that this virus can integrate into the host genome at the AAVS1 gene, no association with human disease has been highlighted thus far [79,80,81,82]. The limited immunogenicity, cytotoxicity, and integration into the host cell genome have made the AAV an ideal delivery system in the gene therapy field (Table 2). Recombinant AAVs have been largely exploited to deliver transgenes of interest into a variety of clinically relevant cells. For example, several natural or variant capsids have been used to deliver the gene encoding the human Factor IX in hemophilia B patients [83,84]. All studies have shown dose-dependent efficacy but also highlighted some immunological responses against the viral capsid [85]. Considering the natural ability of some AAV serotypes to naturally reach the brain, AAV-mediated gene transfer has been attempted to treat neurological defects. Among these, treatment of Parkinson’s disease has been tested in multiple settings [86] and provided significant improvement when AAV2 was used in a clinical trial to deliver the glutamic acid decarboxylase gene to the brain [87]. Similarly, AAVs have shown remarkable efficacy and safety when used to treat ocular degeneration. Clinical exploitation for the treatment of Leber Congenital Amaurosis due to mutations in the *RPE65* gene has been a tremendous success, eventually leading to marketing approval of Luxturna based on the AAV2 vector (Table 3) [88]. A similar path has been followed for the treatment of spinal muscular atrophy, in which the success of AAV9-based gene therapy to replace the defective Survival of Motor Neuron 1 (*SMN1*) gene in motor neurons was ultimately approved as a therapy under the name Zolgensma (Table 3) [89]. Despite the great clinical success of AAV-based gene therapies, the small genome size has limited applications to expression cassettes no larger than 5 kb, leading to many studies that have exploited strategies to deliver larger cargo. Several attempts have been made to split, miniaturize, or create novel synthetic, smaller variants of the transgene of interest to enable AAV-mediated gene transfer with variable success [90]. However, the large amount of knowledge acquired using this vector in over 200 clinical trials has highlighted its remarkable safety and efficacy profiles and confirmed its high potential as a system to deliver gene therapeutics, particularly for in vivo applications. As detailed above, gene therapies based on recombinant AAV vectors have gained marketing approval both in the EU and in the US to treat a variety of human disorders, such as hemophilia A and B, inherited blindness, and spinal muscular atrophy, among others (Table 3). Further studies are certainly necessary to establish concepts for reducing manufacturing costs and to mitigate the AAV-mediated immune response in order to allow more patients with pre-existing immunity to access these innovative therapeutics.

### 2.2. Non-Viral Delivery for Gene Therapy

The use of non-viral strategies to transfer genetic cargo to cells and organisms has gained momentum in the last few years. These include both physical methodologies that exploit different sources of energy (i.e., mechanical, electrical, and sound) to facilitate the penetration of the cargo (i.e., the nucleic acid) through the cellular membrane and non-viral carriers that use molecules capable of forming complexes with the cargo with the double role of protecting it and shuttling it to the cytosol of the target cell via endocytic pathways (Figure 1 and Table 1). Among the most widely used, microinjection, ultrasound, and electroporation are the physical methods that have also been used in clinical settings, while among the non-viral carriers, the use of synthetic lipid nanoparticles or naturally assembled microvesicles certainly provides novel opportunities, particularly for direct in vivo delivery.

#### 2.2.1. Physical Methodologies for Cargo Delivery

Microinjection is a physical delivery method that directly overcomes many cellular barriers by directly injecting the cargo inside the target cells with the use of dedicated equipment. This technique uses a microscope connected to a micromanipulator equipped with a microneedle, through which the nucleic acids are transferred directly into the cytoplasm or nucleus via the application of a gentle pressure. This technique has certainly pioneered the field of genetic engineering, as it was used by Nobel laureate Capecchi to create the first knockout mouse models. As it typically allows the injection of genetic material into a single cell at a time, microinjection is tedious and has typically been applied for the generation of transgenic animals. More recently, this technique has been used to deliver proteins or nucleic acids to difficult-to-reach neurons in vitro. While clinical delivery using this method lags behind, the accurate control over cargo dose and the ability to deliver molecules of any size represent critical advantages that will certainly expand the applicability of this method in the future with parallel developments in the nanotechnologies and microfluidics fields [91].

Ultrasound energy has also been largely used for gene transfer, both in vitro and in vivo. While the exact mechanism is not completely understood, this technique uses ultrasound beams to create local shear forces that in turn generate transient pores in the cellular membrane, allowing diffusion of the cargo to the cytoplasm. The first reports date back to 1991, when this technique was successfully used for plasmid DNA delivery into mammalian cells [92]. Given the large amount of knowledge acquired over decades of using ultrasound-based diagnostics in humans, the application of this concept to facilitate cargo delivery in vivo has made tremendous progress. This has led to seminal studies attempting direct gene delivery in the brains of several animal models, including non-human primates [93,94]. A major advantage of this technique is the substantial specificity that restricts gene expression only to the target region as compared with what is typically achieved when using viral vectors. However, efficiency is typically transient, and the lack of standardization in ultrasound application conditions calls for caution (Table 2). Recently, ultrasounds have been used to safely provide gene therapy delivery through a transient opening of the blood-brain barrier for the treatment of Parkinson disease [95]. Further enhancement of the equipment used might allow effective delivery with reduced ultrasound doses, thus expanding the potential application of this delivery system in humans. It will certainly be interesting to follow the developments of this method, as this might revolutionize accessibility to those tissues, like the brain, that are traditionally resilient to other delivery systems.

Electroporation is a widely used method for physical delivery that involves the application of an electric field around a cell, which ultimately disrupts the structure of the cell membrane, giving rise to the transient formation of pores through which the cargo can diffuse into the cytosol. Typically, the target cells are suspended in an electroconductive buffer in a cuvette among two electrodes. The application of the electric field temporarily disrupts the semi-permeable nature of the cell membrane, allowing macromolecules to permeate the membrane. The effectiveness of electroporation relies on various physical and biological factors, such as the depth of the electric area, the period and frequency of the electric pulse, the cell size, and the cargo concentration. Increasing the duration of the electric pulse generally promotes the creation of bigger pores that remain open for longer periods of time, facilitating cargo delivery. However, it is critical to properly balance the electroporation parameters as membrane disruption, even if transient, leads to major changes in cell homeostasis, electroporation-induced DNA damage, and mitochondrial stress, which can eventually trigger the activation of apoptosis signaling (Table 2). Notwithstanding, electroporation is a relatively simple technique to deliver exogenous molecules into the target cells, and it offers some benefit as compared with viral vectors. The latter are extremely costly, especially when considering clinical-grade manufacturing, while electroporation is a relatively inexpensive method, even though it requires qualified equipment that adheres to good manufacturing practice (GMP) standards, which as of today are limited. Additionally, viral vectors typically have limited cargo capacity, which is typically not the case when using electroporation. However, depending on the target cells, large DNA or RNA molecules might have a reduced efficacy to permeate the target cell membrane, and, particularly when using primary cells, they might induce overt cytotoxicity. Lastly, while some viral vectors have long-term effects due to their integration potential, electroporation can typically be used for transient gene expression, but again, when delivering DNA, it is critical to control its capture in the host genome. It is therefore critical to carefully evaluate which of these two opposing delivery methods might be a better fit for the specific application considered. Seminal studies by Neumann and colleagues used electroporation to deliver plasmid DNA into mammalian cells and provided the first hypothesis on the mechanism that could be mathematically modeled [96]. Subsequently, in the last decades, the refinement of this technology has advanced its use both in basic research and in clinical applications. For example, electroporation has been widely applied in reverse genetics to create disease cellular models [97]. Its flexibility allows the combined delivery of different cargo, which is critical in genome engineering when a DNA repair template is typically co-delivered with a CRISPR-Cas9 ribonucleoprotein for specific editing [98,99]. However, the most exciting application in which electroporation plays a critical role today is the ex vivo manipulation of transplantable primary cells for adoptive cell therapy. Indeed, in a wide variety of clinical applications, genome editing tools have allowed the ex vivo modification of primary T lymphocytes equipped with chimeric antigen receptors or of hematopoietic stem cells that have been then transplanted back to patients to treat specific forms of cancer [100] or a variety of genetic disorders, as recently reported for Sickle Cell Disease (Table 3) [101]. While enhancement of this technique continues to render it more efficient and less disruptive for the target cells, it will be critical to develop novel automated and GMP-compliant devices to render this approach more accessible and facilitate its critical translation.

#### 2.2.2. Non-Viral Carriers for Gene Therapy

Extracellular vesicles (EVs) are classified as exosomes, apoptotic bodies, or microvesicles according to their intracellular origins and size. Among them, exosomes, small vesicles from 30 to 150 nm in length, are released by most cells via the endosomal route and can be found in various biological fluids, including blood plasma, urine, breast milk, and saliva. They carry a diverse range of molecules associated with the pathological and biological states of the original cell, thus serving both as carriers of information from the producer cell and as a delivery system when fusing to target cells. As a consequence, the exosome cargo can be characterized for diagnostic purposes, but it also constitutes a promising tool for the delivery of therapeutic molecules as these remain active during the transfer. In the last decade, tremendous effort has been made to expand the delivery capabilities of EVs, with the goal of packaging therapeutic molecules for their release into target cells. In particular, the field has focused on the delivery of RNA molecules that offer a great deal of flexibility in their application fields, spanning from gene regulation to vaccination and ultimately to gene therapy. However, loading EVs with exogenous RNA is challenging. Typically, this has been achieved by exploiting the natural loading mechanism by which cytoplasmic mature mRNA in high concentrations is naturally packaged in nascent EVs. This is typically achieved by modifying the EV-producer cells accordingly. Less successful has been the electroporation of exogenous mRNA into purified EVs, as this is typically associated with non-reversible membrane disruption. EV loading has not been limited only to mRNA; it has also been exploited to package miRNA, proteins, and DNA. Once loaded with the cargo of choice, EVs typically offer desirable features that render them a valuable candidate for the delivery of gene therapy components. By carefully selecting the origin of the EV-producing cells, the resulting vesicles can be immunologically inert with a beneficial gain in cytotoxicity and can be naturally competent to cross natural biological barriers such as the blood-brain barrier to deliver their cargo to neurons [102]. Furthermore, depending on their membrane composition, these vesicles may have a prolonged half-life when in circulation by evading immune clearance through diverse transmembrane and membrane-anchored proteins [103]. Several groups have attempted to use EV-mediated delivery for therapeutic purposes. Exosomes have been engineered to package gene-editing components, such as the CRISPR-Cas9 system, to excise integrated pathogenic viruses from the genome of infected human cells [104]. For instance, in their 2017 study, Kim et al. described that cancer-derived exosomes loaded with CRISPR/Cas9 plasmids targeting the poly-(ADP-ribose)-polymerase 1 (*PARP-1*) gene cause significant suppression of PARP-1 expression, leading to apoptosis in ovarian cancer cells and enhanced sensitivity to cisplatin. This highlights the potential of these exosomes in cancer therapeutics [105]. On a different note, McAndrews and colleagues explored the use of exosomes as a non-viral delivery system for CRISPR/Cas9 targeting the oncogenic KrasG12D mutation in pancreatic cancer. In this case, exosomes were engineered to package CRISPR/Cas9 plasmids that were successfully delivered to pancreatic cancer cells. This delivery led to targeted gene deletion, inhibition of cell proliferation, and consequently tumor growth in subcutaneous and orthotopic mouse models. Exosomes were characterized for size, the presence of markers, and the efficient delivery of the gene-editing machinery, which shows the potential of being a new platform to achieve CRISPR/Cas9 gene therapy in cancer [106]. While these studies are particularly relevant as exosome uptake from cancer cells is more effective, highlighting a natural and important tropism, direct in vivo injection of engineered EVs has shown limited biodistribution to other organs, such as the liver, lung, and pancreas, that requires proper assessment [107]. In addition, thus far, manufacturing through GMP-compliant procedures lags behind, and a careful characterization and identification of EV cargo is critical to avoid unwanted toxic outcomes and comply with regulatory bodies. Certainly, further enhancement of manufacturing procedures and the use of functional elements to detarget them from unwanted organs may further streamline their applicability in the gene therapy field.

Lipid nanoparticles (LNPs) use positively charged molecules to naturally form compact structures that surround and protect the negatively charged nucleic acids, such as DNA and RNA. Typically, LNPs are lipid-based spherical platforms comprising ionizable cationic lipids, such as 1,2-Dioleoyl-3-trimethylammonium-propane (DOTAP), N-[1-(2,3-Dioleyloxy)propyl]-N,N,N-trimethylammonium (DOTMA), 2,3-dioleyloxy-N-(2-(sperminecarboxamido)ethyl)-N,N-dimethyl-1-propanaminium trifluoroacetate (DOSPA), or Dioleoyl-Glycero-Succinyl-N-Hydroxyethyl Polyamine (DOGS), which are often combined with a neutral lipid, such as 1,2-dioleoyl-sn-glycero-3-phosphoethanolamine (DOPE). The structure is further stabilized by zwitterionic phospholipids and combined with cholesterol to favor adhesion with the target cell membrane and polyethylene glycol (PEG) that covers the outer surface of LNP, thus providing increased stability while decreasing the immune response [108]. Once in contact with the cellular membrane, LNPs are typically endocytosed, and the low pH of this vesicle destabilizes the LNP structure, leading to endosomal escape and cargo release in the cytosol [109]. The efficiency of LNP delivery can be adjusted by varying the concentration and composition of the lipids used for formulation to eventually promote optimal encapsulation, increased stability, and efficient endosomal escape [110]. In the last decade, LNP delivery has been widely used to deliver cytotoxic substances to cancer cells and also to deliver chemotherapeutics. In particular, in the latter application, LNPs have been shown to be particularly effective in reducing the side effects of these drugs by minimizing targeting of normal tissues [111]. Another area where LNP formulations have been instrumental is vaccination. Delivery of mRNA formulated into specific LNPs has shown promising results both to boost T cell response against cancer antigens through specific delivery into dendritic cells [112] and by inducing tolerance to autoantigens by targeting regulatory T cells [113]. More recent is the development of mRNA vaccines against COVID-19 due to the SARS-CoV-2 infection that have been developed at unparalleled speed by Pfizer-BioNTech and Moderna, enabled also by the extensive knowledge on the LNP carrier used (Table 3) [114]. In the field of gene therapy, LNPs have been extensively used to target gene silencing moieties (i.e., siRNA) in different organs, such as the liver or the brain. A seminal study explored the use of LNP-mediated liver delivery of a siRNA targeted to treat transthyretin (TTR)-induced amyloidosis and was the first LNP-siRNA drug to achieve marketing approval in 2018 (Table 3) [115]. More recently, a complementary study successfully achieved a significant decrease in TTR protein serum levels in six patients upon LNP delivery of CRISPR-Cas9 to genetically inactivate the TTR gene [116]. Given the critical interest in genome engineering applications, LNP delivery has focused heavily on the delivery of gene editing components in the last few years. Han and colleagues optimized the delivery of a Cas9 mRNA together with a single guide RNA to target the *antithrombin* (AT) gene in the mouse liver. The efficient suppression of AT led to increased thrombin production and the recovery of bleeding-related symptoms in both hemophilia A and B animals. Significantly, there was no evidence of liver-induced toxicity, unwanted off-target effects, or an immunological response to Cas9, indicating that LNP-mediated CRISPR-Cas9 delivery might be a promising approach to developing novel therapeutics for hemophilia [117]. Further enhancement of LNP formulations has also led to safer and more effective particles for in vivo delivery. By screening a novel library of ionizable lipids, Rosenblum and colleagues identified a novel amino-ionizable lipid that was used to deliver CRISPR-Cas9 components via a single injection directly into the brain of an aggressive glioblastoma mouse model. The authors of this study could show approximately 70% gene editing in vivo at the polo-like kinase 1 (*PLK-1*) target gene, which in turn resulted in tumor cell death, reduced tumor growth, and extended animal survival. Furthermore, to be able to target disseminating tumor cells, the author of the study functionalized the particles using specific antibodies to deliver the *PLK1*-specific CRISPR-Cas9 components to epidermal growth factor receptor (EGFR)-positive tumor cells throughout the animal body, providing a solution to simultaneously target localized and circulating tumors [118]. As LNP delivery systems continue to be enhanced and novel formulations are introduced to improve targeting and biodistribution, we can certainly anticipate growing interest in delivering gene therapeutics using this methodology.

Polyplexes consist of positively charged particles capable of self-assembling through electrostatic interactions with nucleic acids, thus representing a simple carrier for the delivery of DNA or RNA cargo. Among the many polymers available, polyethyleneimine (PEI) [119], poly-aminoacids such as poly-L-lysine [120] or poly-L-ornithine [121], and chitosan [122] are certainly the most widely used. These carriers have the dual role of both protecting the nucleic acids from degradation and interacting with the negatively charged cellular membrane to promote cell entry through endocytosis [123]. Despite the ease of adopting such carriers for the clinical delivery of gene therapy components, the non-trivial degree of cytotoxicity associated with their use represents a critical hurdle (Table 2). For example, the use of PEI has often been associated with overtoxicity, necrosis, and apoptosis [124]. These have been attributed to the electrostatic interaction of the anionic carrier with positively charged proteins, inducing their precipitation, and with the cellular membrane, leading to its destabilization [125]. Similarly, poly-L-lysine was shown to induce critical membrane damage and the loss of cytoplasmic enzymes, such as lactate dehydrogenase, that could be measured in the medium [124]. To mitigate this deleterious effect, researchers have attempted to shield the polymer with moieties that would limit its non-specific interactions. To this end, the use of PEG has been effective in generating more stable polyplex complexes with a significant reduction in cytotoxicity [126]. Furthermore, the exploitation of novel polymers with different structures and properties has also been successfully used to improve the features of existing molecules. In this context, replacing poly-L-lysine with poly-L-ornithine, differing for a single methylene group, resulted in enhanced stability and improved endosomal escape, providing hopes for adoption in gene therapy delivery [121]. Additional modifications of the polymer used can be exploited to improve the stability of the polyplex and the efficiency of specific cell uptake. For example, conjugating PEI with mannose has been successfully explored to specifically deliver the cargo into cells expressing high levels of the mannose receptor, such as dendritic cells [127]. In recent years, polyplexes have also been used to deliver a variety of components for genome editing applications, such as plasmid DNA, mRNA, or CRISPR-Cas9 ribonucleoproteins. Wang and colleagues reported on the development of a novel redox-responsive cationic polymer that can effectively deliver a variety of negatively charged cargo with undetectable toxicity in cell lines [128]. These results are certainly encouraging and promote the use of polyplexes as an adaptable and promising mode for delivering gene therapy components in a non-viral manner. Certainly, to estimate their full clinical potential, further work is necessary to assess efficiency and cytotoxicity when applying them to primary transplantable human cells, which are typically less resilient to manipulation.

Polyplex micelles have acquired a lot of attention for their potential in gene editing applications. They are typically considered virus-like structures as they mimic viral features that confer improved shielding to the cargo and are therefore well-suited to deliver highly sensitive molecules such as mRNA. In this carrier, the use of block copolymers leads to condensation of the nucleic acid cargo in a core-shell structure surrounded by hydrophilic components, often PEG. This architecture is able to completely block the access of external enzymes, such as nucleases, to the cargo with a critical gain in stability [129]. In the field of targeted gene therapeutics, polyplex micelles have exhibited enormous potential to deliver different types of gene therapeutics directly in vivo. A structurally optimized polyplex micelle formulation was developed by Dirisala and colleagues to deliver an antiangiogenic factor in a mouse model of human pancreatic adenocarcinoma. The authors reported a significant inhibition of tumor growth upon intravenous administration, highlighting that structural optimization of the polymers used is critical to achieving efficient cargo delivery, in particular for systemic applications [130]. Furthermore, polyplex micelles have also been successfully used to deliver genome editing components for achieving precise genetic changes in vivo. In this case, PEG-ylated micelles were used to stabilize the Cas9-encoding mRNA with a guide RNA targeting a reporter gene cassette. Upon direct injection into the murine brain, Abbasi and colleagues demonstrated efficient reporter gene inactivation in murine brain cells, including neurons, astrocytes, and microglia [131]. Taken together, these data clearly show the translation potential of polyplex micelles that can be efficiently used as carriers for gene therapies in vivo. Formulation is certainly critical, and further development in that direction will likely provide novel polymers with improved efficacy to safely deliver gene therapeutics.

## 3. Conclusions

Progress in gene therapy and in the genome engineering field has made tremendous advances in the last twenty years. The efforts of many researchers worldwide have eventually transformed the concept of curing a disease with genes into a clinical reality. Instrumental to this success has been the enhancement of more efficient and safer delivery systems to release the necessary components into the target cell or tissue. While new platforms have emerged in rapid succession, many challenges have to still be addressed to ensure efficient delivery of gene therapeutics and, ultimately, enable safe treatment. The non-specific clearance of viral and non-viral delivery carriers is a major obstacle to the clinical translation of gene therapies. This issue drastically reduces delivery efficiency to target tissues beyond the liver and often provokes toxicity concerns (Table 2). Liver sinusoidal accumulation has been documented for different viral vectors, including AdV, and for lipid nanoparticles [132]. Strategies aimed at mitigating non-specific liver clearance are of great significance for improving the efficacy and safety of gene delivery systems. Several strategies have been pursued toward this goal. One of them is the transient coating of sinusoidal wall cells with biocompatible materials, such as two-arm PEG-OligoLysine, that prevents the clearance of both non-viral and viral gene vectors. This coating temporarily masks vectors from the liver’s phagocytic cells, thereby reducing their uptake and increasing their circulation time. As a consequence, a greater proportion of the vectors can reach the intended target tissues [133]. Also, transient depletion of Kupffer cells prior to the administration of gene therapeutics significantly reduces the liver accumulation of these carriers [134]. This approach allows more of the therapeutic payload to evade hepatic capture and reach the target tissues. To avoid liver targeting, an alternative strategy has been developed in which the outer surface of the carrier is modified with ligands that bind preferentially to receptors present on the target tissue or cell [135]. For example, the inclusion of targeting ligands such as the arginylglycylaspartic acid (RGD) peptide ensures that a larger fraction of carriers is delivered to tumor cells rather than to the liver [136]. By employing these approaches, the non-specific clearance of gene delivery carriers by the liver can be minimized, thereby improving the delivery efficiency to target tissues and reducing toxicity concerns.

The different systems described above are complementary tools that enable the development of gene therapeutics in different conditions and that serve different purposes, spanning from gene complementation to vaccination. In an era where the success of multiple clinical trials has bolstered the use of novel gene therapeutics to improve patient life (Table 3), further developments are certainly necessary to enhance biocompatibility and tissue targeting. This will facilitate direct in vivo delivery, which will ultimately help to significantly reduce manufacturing costs, thereby facilitating their widespread applicability and patient access to these life-changing therapies.

## Figures and Tables

**Table 1:** Delivery Methods for Gene Editing Components.

Delivery Method	Type	Genes Delivered	Target Cells/Tissues
Gamma Retrovirus	Viral	DNA, shRNA	Hematopoietic cells, T cells, stem cells [15,16]
Lentivirus	Viral	DNA, shRNA, CRISPR-Cas9 components	Hematopoietic cells, stem cells, T cells, neurons [17,18,19]
Adenovirus	Viral	DNA, RNA, CRISPR-Cas9 components	Respiratory epithelial cells, liver cells, muscle cells, cancer cells [20,21,22]
Adeno-Associated Virus (AAV)	Viral	DNA, CRISPR-Cas9 components	Liver cells, muscle cells, neurons, retinal cells [23]
Extracellular Vesicles (EVs)	Non-Viral	mRNA, miRNA, siRNA, DNA	Immune cells, cancer cells, neurons, stem cells [24]
Lipid Nanoparticles (LNPs)	Non-Viral	mRNA, siRNA, CRISPR-Cas9 components	Liver cells, muscle cells, lung cells, tumor cells [25,26]
Polyplex	Non-Viral	DNA, RNA	Various cell types including tumor cells, liver cells [27]
Polyplex Micelles	Non-Viral	DNA, RNA	Tumor cells, immune cells [28]
Ultrasound	Physical	DNA, RNA, CRISPR-Cas9 components	Tumor cells, localized tissues (e.g., liver, muscle) [29]
Microinjection	Physical	DNA, RNA, CRISPR-Cas9 components	Oocytes, zygotes, embryos, single cells [30]
Electroporation	Physical	DNA, RNA, CRISPR-Cas9 components	Various cell types including muscle cells, immune cells, cancer cells [31]

**Table 2:** Limitations of Viral, Non-Viral Gene Delivery Carriers, and Physical Methods [38,39].

Delivery Method	Type	Limitations
Gamma Retrovirus	Viral	Integration into host genome causing mutagenesis, immunogenicity, limited to dividing cells
Lentivirus	Viral	Integration into host genome causing mutagenesis, immunogenicity, production complexity
Adenovirus	Viral	Strong immune response, transient expression, potential toxicity
Adeno-Associated Virus (AAV)	Viral	Limited cargo size, potential for immune response, high production cost
Extracellular Vesicles (EVs)	Non-Viral	Heterogeneity, low yield, potential for immune response, scalability issues
Lipid Nanoparticles (LNPs)	Non-Viral	Potential toxicity, instability, limited targeting specificity
Polyplex	Non-Viral	Potential cytotoxicity, low transfection efficiency, instability in biological fluids
Polyplex Micelles	Non-Viral	Low transfection efficiency, potential cytotoxicity, stability issues
Ultrasound	Physical	Limited tissue penetration, potential for tissue damage, requires specialized equipment
Microinjection	Physical	Labor-intensive, low throughput, potential for cell damage
Electroporation	Physical	Potential for cell damage, low efficiency in some cell types, requires specialized equipment

**Table 3:** FDA and EMA-Approved Gene Delivery Carriers and Methods [40,41,42,43].

Gene Delivery Carrier	Type	FDA Approval Year	Application/Indication
Glybera (Alipogene tiparvovec)	Viral (Adeno-Associated Virus—AAV)	2012 (EU, not FDA)	Lipoprotein lipase deficiency
Luxturna (Voretigene neparvovec)	Viral (AAV)	2017	Inherited retinal dystrophy
Strimvelis	Viral (retrovirus)	2016	ADA-SCID
Zolgensma (Onasemnogene abeparvovec)	Viral (AAV)	2019	Spinal muscular atrophy
Imlygic (Talimogene laherparepvec)	Viral (Modified Herpes Simplex Virus)	2015	Melanoma
Kymriah (Tisagenlecleucel)	Viral (Lentivirus)	2017	B-cell acute lymphoblastic leukemia
Yescarta (Axicabtagene ciloleucel)	Viral (Lentivirus)	2017	Large B-cell lymphoma
Zynteglo (Betibeglogene autotemcel)	Viral (Lentivirus)	2019 (EU, 2022 FDA)	Beta-thalassemia
Breyanzi (Lisocabtagene maraleucel)	Viral (Lentivirus)	2021	Large B-cell lymphoma
Abecma (Idecabtagene vicleucel)	Viral (Lentivirus)	2021	Multiple myeloma
Onpattro (Patisiran)	Non-Viral (Lipid Nanoparticles—LNPs)	2018	Hereditary transthyretin-mediated amyloidosis
Pfizer-BioNTech COVID-19 Vaccine (BNT162b2),	Non-Viral (Lipid Nanoparticles—LNPs)	2020	COVID-19 Vaccination
Moderna COVID-19 Vaccine (mRNA-1273)	Non-Viral (Lipid Nanoparticles—LNPs)	2020	COVID-19 Vaccination
Bleomycin (with Electroporation)	Physical (Electroporation)	Not specified (approved for cancer treatment)	Various cancers, especially head and neck cancer

## Data Availability

No new data were created or analyzed in this study. Data sharing is not applicable to this article.
